# Feasibility of very short-term forecast models for COVID-19 hospital-based surveillance

**DOI:** 10.1590/0037-8682-0762-2020

**Published:** 2021-02-10

**Authors:** Edson Zangiacomi Martinez, Afonso Dinis Costa Passos, Antônio Fernando Cinto, Andreia Cássia Escarso, Rosane Aparecida Monteiro, Jorgete Maria e Silva, Fernando Bellissimo-Rodrigues, Davi Casale Aragon

**Affiliations:** 1 Universidade de São Paulo, Faculdade de Medicina de Ribeirão Preto, Ribeirão Preto, SP, Brasil.; 2 Universidade de São Paulo, Faculdade de Medicina de Ribeirão Preto, Núcleo de Vigilância Epidemiológica Hospitalar, Ribeirão Preto, SP, Brasil.

**Keywords:** COVID-19, Coronavirus disease, Forecasting, Statistical models, Epidemiology

## Abstract

**INTRODUCTION:**

We evaluated the performance of Bayesian vector autoregressive (BVAR) and Holt’s models to forecast the weekly COVID-19 reported cases in six units of a large hospital.

**METHODS:**

Cases reported from epidemiologic weeks (EW) 12-37 were selected as the training period, and from EW 38-41 as the test period.

**RESULTS:**

The models performed well in forecasting cases within one or two weeks following the end of the time-series, but forecasts for a more distant period were inaccurate.

**CONCLUSIONS:**

Both models offered reasonable performance in very short-term forecasts for confirmed cases of COVID-19.

The coronavirus disease (COVID-19) pandemic has already claimed more than 190,000 lives in Brazil at the time of this writing. Among the many efforts made to increase knowledge about this new disease and enable strategies for its mitigation, mathematical and statistical models can provide useful information about the dynamics of COVID-19. These approaches include susceptible-infected-recovered (SIR) models and their extensions, exponential smoothing, and models based on S-shaped curves, such as the logistic, Gompertz, and Richards curves^1^. These models are used to describe the temporal variations of an outbreak and can provide short-term forecasts of new cases of the disease. Furthermore, when the aim is to evaluate two or more simultaneous time-series of events, we can consider the use of multivariate approaches such as vector autoregressive (VAR) models^2^. VAR models are very useful, especially in the field of economics^2^, but they also have applications in epidemiology^3^, including the modeling of COVID-19 data^4^.

In this study, we used a multivariate time-series analysis based on a Bayesian extension of the VAR model in order to describe the weekly number of confirmed cases of COVID-19 reported in six units of the Clinical Hospital of the Ribeirão Preto Medical School (HCFMRP), University of São Paulo (USP), and to explore the possibility of very short-term forecasts. Multivariate time-series data can provide more information than univariate time-series data. VAR models and their extensions allow us to investigate how each variable impacts the other in each separate time-series, and this can improve the forecasting process. We compared the forecast accuracy of this multivariate analysis with those obtained by exponential smoothing based on Holt's method.

We used data from the Center of Epidemiological and Hospital Surveillance (NVEH) of the HCFMRP. By convention, epidemiological weeks (EW) run from Sunday to Saturday, and we considered the weekly number of confirmed cases of COVID-19 reported from EW 12 (March 15-21, 2020) to EW 41 (October 4-10, 2020). The HCFMRP is a large hospital complex with many health departments and 1,194 general beds in total. There were 45,866 hospitalizations and 41,377 surgeries in 2019. The health units of the HCFMRP included in the present study were the central unit located on the USP campus (here referred as "Campus"), the State Hospital of Américo Brasiliense (HEAB), the State Hospital of Ribeirão Preto (HERP), the State Hospital of Serrana (HES), the Reference Center in Women's Health (MATER), and the Emergency Unity (UE). Four of these units are located in Ribeirão Preto, a medium-sized city in the northwest region of the state of São Paulo, Brazil, with approximately 700,000 inhabitants, and the other two units are located in the cities of Américo Brasiliense and Serrana, located near Ribeirão Preto. Patients suspected of infection were tested for COVID-19 by reverse transcription polymerase chain reaction (RT-PCR). This was a study conducted with anonymized secondary data and therefore did not require approval from the Human Research Ethics Committee.

Formally, a time-series is defined as a collection of random variables {*Y*_*t*_ }, where the time index *t* assumes integer values *t* = 1, 2, 3, and so on. Let us consider a K-dimensional multivariate time-series denoted by Y_*t*_ = (*Y*_1t_, …, *Y*_*Kt*_ ). The VAR model, in this matrix form, is given by $Y_{t}=B_{0}+B_{1}Y_{t-1}+B_{2}Y_{t-2}+\ldots +B_{2}Y_{t-p}+e_{t}$, where *p* is the order of the autoregressive process (lag), B_0_ = (*b*_10_, *b*_20_, ..., *b*_*K0*_ ) is a K×1 column vector of coefficients, B_*j*_ are KxK matrices of coefficients (*j* = 1,...,*p*), and ***e***_*t*_ = (*e*_*1(t)*_ , *e*_*2(t)*_ , ..., *e*_*K(t)*_ ) are the errors of the model^2^ ^,^ ^3^. It is often assumed that the errors follow a multivariate normal distribution with a vector mean of 0 and variance-covariance matrix **S**. An autoregressive model of order *p* was used, meaning that the *p* preceding values are used to predict the next value. In this study, we consider a multivariate set of K = 6 time series, each relative to a health unit of HCFMRP. The length of each series was 30 weeks, considering data until October 10, 2020. The estimation of a VAR model uses an equation-by-equation approach, where ordinary least squares (OLS) regressions were used for each equation. One of the prerequisites for the estimation of a VAR model is that the analyzed time-series are stationary, or that they show similar behavior throughout their duration. In this case, their mean and variance remained unchanged with time. Brooks^5^ argues that, “*if one wishes to use hypothesis tests, either singly or jointly, to examine the statistical significance of the coefficients, then it is essential that all of the components in the VAR are stationary.*” As we used the cumulative number of COVID-19 cases to conduct model fitting, variables are obviously non-stationary given the increasing behavior of the corresponding curves. As an alternative, we can use the Bayesian vector autoregressive (BVAR) model. BVAR models use Bayesian methods to estimate the parameters of the VAR model, and according to Holden^6^, some authors have argued that this approach may be applied to non-stationary variables. For example, in a study discussing the asymptotic distribution theory for statistics from autoregressive models with a unit root, including VAR models, Sims et al.^7^ claim that “*because the Bayesian approach is entirely based on the likelihood function, which has the same Gaussian shape regardless of the presence of nonstationarity, Bayesian inference need take no special account of nonstationarity*.” Gupta and Kotzé^8^ also provide some discussion on this.

The Akaike information criterion (AIC) values for different models with lag length (*p*) ranging from 1 to 6 are, respectively, given by 969.8, 898.6, 880.2, 761.5, 626.4, and 839.9. Models with *p* > 6 were not fitted, because high-order lags may overfit the data. Based on these AIC values, we decided to consider *p* = 5, because models with lower AIC values are preferred. Figure 1 describes the time-series for the cumulative number of cases reported in each health unit and the predicted values obtained from the BVAR model, based on *p* = 1 and *p* = 5. These models were fitted using the Metropolis-Hastings algorithm in the R package “BVAR”, based on 100,000 simulated samples taken after discarding an initial 5,000 burn-in period. Convergence of the simulated samples was verified by trace and density plots, and correlation between successive samples was inspected with autocorrelation plots. The BVAR package uses the Minnesota prior as baseline^9^, which is a prior distribution that transforms the VAR model into a random walk process for each variable.

Figure 1 shows a sudden increase in the number of hospitalizations at the HERP in EW 31, which is accompanied by a decrease in the number of hospitalizations in the other units with the most beds. This decrease is not so clear in the corresponding graphs because the cumulative number of hospitalizations is relatively large in these units, but this situation describes the possible reallocations of patients between the units of HCFMRP. In this way, when considering interrelations between time-series variables, the BVAR model can provide an effective method to cope with these effects.

**FIGURE 1:**
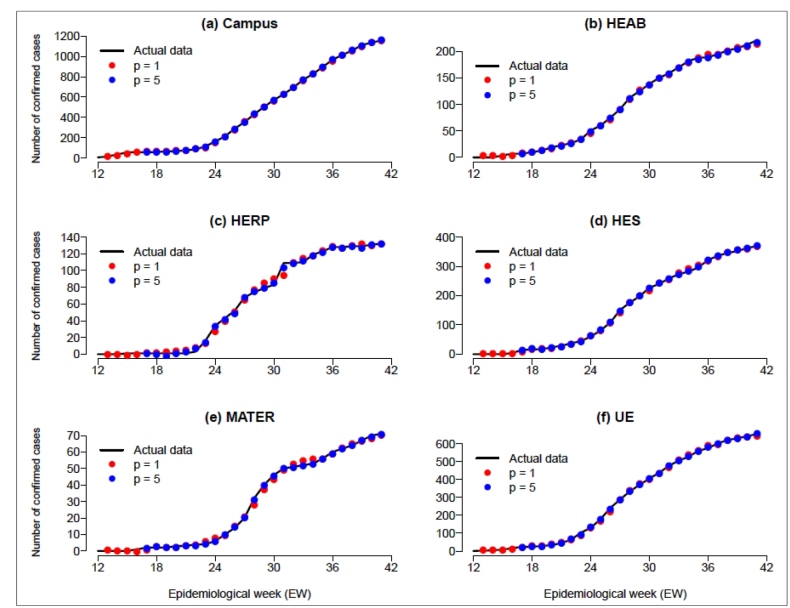
Cumulative number of COVID-19 cases in each health unit of the HCFMRP, and fitted values obtained from the BVAR model based on lags of length p = 1 and p = 5. **HCFMRP:** Clinical Hospital of the Ribeirão Preto Medical School; **BVAR:** Bayesian vector autoregressive; **HEAB:** State Hospital of Américo Brasiliense; **HERP:** State Hospital of Ribeirão Preto; **HES:** State Hospital of Serrana; **MATER:** Reference Center in Women's Health; **UE:** Emergency Unity.

Holt's method is a special case of the Holt-Winters exponential model in which seasonality is absent. In the equation for Holt’s method, the forecasted value of the series at time *t* is given by

$${\hat{Y}}_{t}=L_{t-1}+T_{t-1},$$

where *L*_*t*_ is the estimated level given by

$$L_{t}=\alpha Y_{t}+\left(1-\alpha \right)\left(L_{t-1}+T_{t-1}\right),$$

*T*_*t*_ is the estimated slope given by

$$T_{t}=\beta \left(L_{t}-L_{t-1}\right)+\left(1-\beta \right)T_{t-1},$$

and α and β are smoothing parameters^10^ ^,^ ^11^. Holt's model can be applied by using the “holt” function included in the R package “forecast”.

We then assessed the feasibility of using BVAR and Holt’s models to obtain short-term forecasts for weekly COVID-19 cases in each health unit. Given that Holt's method is a univariate approach, we adjusted independent models for each health unit. We considered weekly reports from the date on which the first case was notified in one of the units of HCFMRP up to EW 37 as the training period. The values of the validation period were the corresponding observations from EW 38-41. Comparisons between the forecasted and actual values were based on the mean absolute percent error (MAPE), and Theil's U entropy coefficient was used as a measure of out-of-sample forecasting accuracy^12^. The lower the MAPE value, the better the performance of the corresponding model. When Theil's coefficient is greater than 1, the forecasts under consideration are less accurate than those obtained by a naïve approach, or a simple method in which the forecasts are equal to the last observed value.

Table 1 shows the weekly observed COVID-19 cases and the corresponding forecasts obtained from the BVAR and Holt’s models (with 95% prediction intervals) in the validation period. Table 2 shows the MAPE and Theil's U values considering forecasts of length h = 3 and h = 4 weeks. The BVAR model appears to perform better in terms of MAPE in some health units, such as Campus and HES, while the univariate Holt’s model appears to perform better in other units. However, in a general manner, both models are inefficient in making forecasts over periods longer than 2 weeks, given that Theil's U values are higher than 1 for some health units considering forecasts of length h = 3 or higher (Table 2). In the context of our study, this allows us to conclude that both BVAR and Holt’s models are useful for predicting the future number of confirmed cases of COVID-19 in the week following the end of an observed time-series, but forecasts for a more distant period can be very inaccurate.

**TABLE 1:** Weekly COVID-19 cases and the corresponding forecasts from the BVAR and Holt’s models (with 95% prediction intervals), from epidemiological weeks 38-41.

			Forecasted values			
Unit	EW	Observed	Holt’s	95% prediction	BVAR	95% prediction
		values	model	interval	model	interval
						
**Campus**	38	1059	1069.48	1043.28 - 1095.69	1070.39	1041.19 - 1098.77
	39	1114	1120.44	1067.15 - 1173.73	1110.18	1054.77 - 1164.98
	40	1137	1171.40	1083.74 - 1259.05	1140.00	1040.54 - 1242.47
	41	1160	1222.35	1094.71 - 1349.99	1161.54	1000.19 - 1336.78
						
**HEAB**	38	202	199.33	190.73 - 207.94	202.79	194.50 - 211.39
	39	206	203.87	188.02 - 219.72	206.90	193.01 - 222.60
	40	213	208.41	183.78 - 233.03	205.91	182.22 - 233.93
	41	221	212.94	178.29 - 247.59	201.09	164.04 - 249.16
						
**HERP**	38	129	132.35	118.51 - 146.19	129.09	107.84 - 149.97
	39	129	136.54	114.26 - 158.82	125.06	88.25 - 160.49
	40	131	140.74	109.81 - 171.66	122.31	71.66 - 171.54
	41	132	144.93	104.88 - 184.97	122.91	60.88 - 184.57
						
**HES**	38	346	353.36	337.48 - 369.23	344.94	326.52 - 362.70
	39	352	370.33	339.88 - 400.78	355.94	327.11 - 386.78
	40	363	387.30	339.43 - 435.17	351.47	311.42 - 400.59
	41	369	404.28	336.56 - 471.98	354.21	297.34 - 430.11
						
**MATER**	38	64	64.00	59.86 - 68.14	64.19	59.03 - 69.40
	39	67	66.00	56.74 - 75.26	67.36	58.17 - 75.84
	40	70	68.00	52.51 - 83.49	68.72	57.61 - 79.06
	41	71	70.00	47.33 - 92.67	65.45	53.54 - 78.87
						
**UE**	38	620	624.59	604.38 - 644.79	619.40	579.59 - 657.27
	39	631	648.21	609.41 - 687.01	610.16	546.82 - 674.33
	40	641	671.84	608.80 - 734.87	611.37	516.27 - 720.06
	41	655	695.46	604.03 - 786.89	614.69	464.72 - 790.30
						

**EW:** epidemiological week; **BVAR:** Bayesian vector autoregressive; **HEAB:** State Hospital of Américo Brasiliense; **HERP:** State Hospital of Ribeirão Preto; **HES:** State Hospital of Serrana; **MATER:** Reference Center in Women's Health; **UE:** Emergency Unity.

**TABLE 2:** Mean absolute percent errors (MAPE) and Theil's U entropy coefficients for forecasting weekly COVID-19 cases in each health unit of the HCFMRP, considering forecasts of length h = 3 and h = 4.

		Holt’s model		BVAR model	
Unit	Length of forecast	MAPE	Theil’s U	MAPE	Theil’s U
					
**Campus**	h = 3	1.531	0.563	0.561	0.081
	h = 4	2.492	1.063	0.454	0.079
					
**HEAB**	h = 3	1.503	0.672	1.386	0.882
	h = 4	2.039	0.831	3.291	1.833
					
**HERP**	h = 3	5.292	6.157	3.252	4.771
	h = 4	6.417	7.946	4.161	5.869
					
**HES**	h = 3	4.677	2.435	1.534	0.970
	h = 4	5.898	3.313	2.152	1.359
					
**MATER**	h = 3	1.449	0.519	0.888	0.307
	h = 4	1.439	0.551	2.620	1.231
					
**UE**	h = 3	2.760	2.363	2.674	2.427
	h = 4	3.614	2.617	3.544	2.643
					

**HCFMRP:** Clinical Hospital of the Ribeirão Preto Medical School; **MAPE:** Mean absolute percent error; **BVAR:** Bayesian vector autoregressive; **HEAB:** State Hospital of Américo Brasiliense; **HERP:** State Hospital of Ribeirão Preto; **HES:** State Hospital of Serrana; **MATER:** Reference Center in Women's Health; **UE:** Emergency Unity.

Even in a hospital context, the dynamics of COVID-19 can change very quickly during the time course of disease in response to a large number of factors, including changes in mitigation strategies for transmission in the community and people's adherence to them, and the availability of tests for essential screening^13^. The future of an epidemic in a population during its course is thus hard to predict, and mathematical and statistical models are only capable of simulating what can happen in the future if the conditions observed in the present do not change^14^. In the present study, we have data reported in weeks, and a period of two weeks or more can be large enough to produce substantial changes in exogenous variables that affect the dynamics of the disease. Although important, these limitations do not reduce the usefulness of the study in obtaining very short-term forecasts for confirmed cases of COVID-19, since out-of-sample predictions for the next one or two weeks can provide useful information for healthcare planning and distribution of resources. In economics, very short-term forecasting is sometimes called nowcasting (a contraction of "now" and "forecasting"), where forecasts for a very near future are useful to monitor changes in variables of interest in real time^15^. In the context of the HCFMRP, accurate short-term forecasts can create alerts for situations of peak demand for hospitalizations during the pandemic and can help health managers optimize the costs of supplies and staff. Our results suggest that the BVAR model is a feasible tool to obtain these short-term forecasts. In addition, the “BVAR” package in R offers researchers with programming knowledge a powerful tool for the study of multivariate time-series in epidemiology.

The limitations of the present study include uncertainties in the diagnostic accuracy of the tests used to determine the COVID-19 status and, from a statistical perspective, the fact that the time-series are relatively short and the weekly case counts were sometimes small, which can make inferences difficult. Despite these shortcomings, both BVAR and Holt’s models offered reasonable performances in very short-term forecasts for confirmed cases of COVID-19 and can be valuable tools for disease surveillance.
